# Evolving Diagnostic and Treatment Strategies for Pancreatic Neuroendocrine Tumors

**DOI:** 10.1186/1756-8722-4-29

**Published:** 2011-06-14

**Authors:** Matthew H Kulke, Johanna Bendell, Larry Kvols, Joel Picus, Rodney Pommier, James Yao

**Affiliations:** 1 Dana-Farber Cancer Institute, Boston MA, USA; 2 Sarah Cannon Research Institute, Nashville, TN, USA; 3 H Lee Moffitt Cancer Center, Tampa FL, USA; 4 Siteman Cancer Center, St Louis MO, USA; 5 Oregon Health and Science University, Portland OR, USA; 6 MD Anderson Cancer Center, Houston TX, USA

## Abstract

Pancreatic neuroendocrine tumors (NET) have diverse clinical presentations. Patients with symptoms of hormone secretion may require specific medical interventions to control those symptoms prior to antitumor intervention. In some patients, tumors in the pancreas may be occult and specialized diagnostic imaging or surgery may be required for diagnosis. Other patients may present with more advanced disease, presenting with symptoms of tumor bulk rather than hormone secretion. Treatment options for patients with advanced pancreatic neuroendocrine tumors include surgical resection and hepatic directed therapies, including partial hepatectomy, hepatic artery embolization, or other ablative techniques. Streptozocin or temozolomide-based chemotherapy regimens are active against pancreatic NET, and can also play an important role in the palliation of patients with advanced disease. A number of biologically targeted agents targeting the VEGF and mTOR signaling pathways have recently shown promise, with recent trials showing treatment with the VEGFR tyrosine kinase inhibitor sunitinib or the mTOR inhibitor everolimus improves progression-free survival in patients with advanced NET.

## Introduction

Pancreatic neuroendocrine tumors (NET) have been considered rare, with an estimated incidence of less than 1 per 100,000 individuals [1]. In recent years, however, the diagnosed incidence of pancreatic NET has increased, an observation that is likely due, at least in part, to improved detection and classification [2]. The diverse and sometimes non-specific clinical syndromes associated with pancreatic NET can make these malignancies difficult to diagnose at an early stage. Awareness of the clinical presentation and treatment options for patients with pancreatic NET has become increasingly relevant for both medical oncologists and other health care providers, as new treatment options emerge for patients with this disease.

## Histologic Classification and Staging

Pancreatic NET have also been referred to as pancreatic islet cell tumors or pancreatic endocrine tumors. Carcinoid tumors have a similar histologic appearance to pancreatic NET, but generally arise in the bronchi, small intestine, appendix, or rectum. While the term "pancreatic carcinoid" has also sometimes been used to describe pancreatic NET, this term is considered confusing as the clinical presentation and treatment options for pancreatic NET differ in many respects from those for carcinoid tumors.

The majority of pancreatic NET occur sporadically. However, pancreatic NET can be associated with inherited genetic syndromes; in particular, approximately 10% may be associated with multiple endocrine neoplasia type 1 (MEN1). MEN1 is an autosomal dominant syndrome associated with mutations in the tumor suppressor gene *menin*, and is characterized by the development of multiple NET involving not only the pancreas but also the parathyroid and pituitary glands [3]. Pancreatic NET have also been associated with MEN2, Von Hippel-Lindau disease, tuberous sclerosis, and neurofibromatosis.

The histologic features of pancreatic NET can vary, affecting both prognosis and treatment recommendations. An important first step following the diagnosis of a pancreatic malignancy is the differentiation of neuroendocrine cancers from the far more common pancreatic adenocarcinoma. Though the pathologic criteria for differentiating these two entities are clear, limited tissue from fine needle aspirations or endoscopic brushings may preclude accurate diagnosis. In questionable cases, repeat tissue sampling should be performed, particularly if systemic treatments are being considered.

Adequate tissue sampling is also critical in differentiating the various subtypes of pancreatic NET. These tumors may fall within a broad spectrum of well-differentiated, low grade tumors to more poorly differentiated, high grade tumors. While a number of histologic classification systems have been proposed for pancreatic NET, tumors with a mitotic count >20/10 high powered fields or a Ki-67 proliferation index of >20% generally represent highly aggressive malignancies where treatment with platinum based regimens is generally indicated, according to small cell carcinoma guidelines [4,5].

The American Joint Committee on Cancer (AJCC) staging system for pancreatic NET is increasingly accepted as the standard staging system in North America, and is similar to the system used for pancreatic adenocarcinomas. Several other organizations, including both North-American based groups and the European Neuroendocrine Tumor Society (ENETs) have proposed similar, though not identical, staging systems for NET using the commonly accepted Tumor Node Metastasis (TNM) notation [6-10].

## Clinical Presentation and Initial Management

Most pancreatic NET are considered "non-functional" in that they are not associated with symptoms of hormone hypersecretion. Such tumors are usually identified incidentally during imaging for other indications, or at an advanced stage, when patients become symptomatic from tumor bulk. Patients with hormonal hypersecretion, on the other hand, can present with diverse and sometimes puzzling clinical symptoms (Table 1). Specific recommendations for some of the more common tumors, based on the clinical presentation and hormones secreted, are described below.

**Table 1:** Clinical presentation of pancreatic neuroendocrine tumors (NET)

Tumor	Symptoms or signs	Incidence of metastases	Extrapancreatic location
Insulinoma	Hypoglycemia resulting in intermittent confusion, sweating, weakness, nausea; loss of consciousness may occur in severe cases	<15%	Rare

Glucagonoma	Rash (necrotizing migratory erythema), cachexia, diabetes, deep venous thrombosis	Majority	Rare

VIPoma, Verner-Morrison Syndrome, WDHA Syndrome	Profound secretory diarrhea, electrolyte disturbances	Majority	10%

Gastrinoma, Zollinger-Ellison Syndrome	Acid hypersecretion resulting in refractory peptic ulcer disease, abdominal pain, and diarrhea	<50%	Frequently in duodenum

Somatostatinoma	Diabetes, diarrhea, cholelithiasis	Majority	Rare

Non-functioning	May be first diagnosed due to mass effect	Majority	Rare

WDHA: Watery Diarrhea, Hypokalemia and Achlorhydria.

### Insulinoma

Insulinomas classically present with "Whipple's Triad:" a combination of symptoms of hypoglycemia, inappropriately high insulin levels with associated documented blood glucose levels of <50 mg/dL, and symptom relief with administration of glucose [11]. Initially, the hypoglycemia may be managed with dietary modifications or with diazoxide [12]. For these patients, octreotide or other somatostatin analogs should be used with caution, as they have the potential to worsen hypoglycemia by suppressing glucagon secretion. Treatment with the mTOR inhibitor everolimus has also been reported to be beneficial in insulinoma patients with refractory hypoglycemia [13].

### Glucagonoma

Over two-thirds of patients with glucagonomas present with necrolytic migratory erythema, a rash characterized by raised erythematous patches beginning in the perineum and progressing to the trunk and extremities [14,15]. Somatostatin analogs are generally successful in the initial management of patients with the glucagonoma syndrome [16,17]. Glucagonomas may be associated with diabetes mellitus, though only half of patients experience clinically significant hyperglycemia.

### Gastrinoma and Zollinger-Ellison syndrome

The gastrinoma syndrome is characterized by gastric hypersecretion [18]. In patients with non-healing peptic ulcers and a fasting gastrin level >100 pg/mL, a diagnosis of gastrinoma should be considered [19]. Moderate elevations of serum gastrin may also, however, be seen in patients receiving concomitant therapy with proton pump inhibitors, sometimes complicating efforts to confirm a diagnosis. Proton pump inhibitors are a highly effective initial treatment in controlling symptoms associated with gastric hypersecretion [20,21]. Treatment with somatostatin analogs has also been associated with improved control of serum gastrin levels and, in some cases, with tumor stabilization or regression [22].

### VIPoma

Pancreatic endocrine tumors associated with profound diarrhea, hypokalemia, and achlorhydria were first described by Verner and Morrison in 1958 [23]. This syndrome was subsequently found to be due to ectopic vasoactive intestinal peptide (VIP) secretion. Treatment with somatostatin analogs is effective in treatment of diarrhea in these patients [24].

## Imaging

Patients with functioning tumors, particularly insulinomas and gastrinomas, may develop hormonal symptoms from small primary tumors, and localization of the primary lesions may be challenging. Traditional cross-sectional imaging with triple phase CT or MRI is generally the first step in attempting to localize these tumors. Endoscopic ultrasound may be more sensitive than CT or MRI for the detection of small lesions, and may also provide useful information regarding potential vessel involvement prior to planned resection. Pancreatic NET, like carcinoid tumors, frequently over express somatostatin receptors. ^111^Indium-DTPA-octreotide (Octreoscan™) has been commonly used, often in combination with cross-sectional imaging, to localize and stage pancreatic NET.

## Biochemical Assessment and Monitoring

In patients with symptoms of hormone hypersecretion, serial measurements of the specific hormone may be helpful in assessing treatment response or in monitoring for recurrence. The majority of patients with pancreatic NET, however, do not have clear evidence of hormone hypersecretion. Serum chromogranin A (CGA) is a neuroendocrine secretory protein that serves as a marker of disease activity in both functional and non-functional pancreatic NET [1,25-27]. CGA may decrease in patients responding to somatostatin analogs or other therapies [26,28]. In patients on stable SSA doses, consistent increases in plasma CGA levels over time may reflect loss of secretory control and/or tumor growth [25-27,29,30]. Use of CGA as a diagnostic or screening test for pancreatic NET is discouraged, as CGA may be elevated in a number of non-malignant conditions, including renal insufficiency and liver disease, and in patients taking proton pump inhibitors.

## Surgical Management

**Figure 1:**
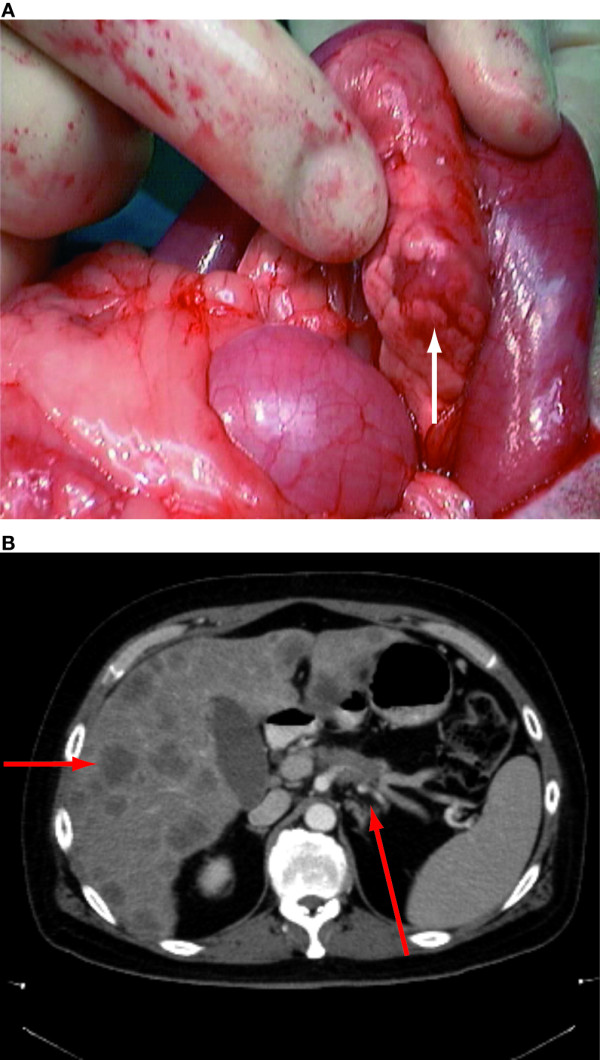
**Variations in size of primary pancreatic NET**. A: Insulinoma presenting as an occult nodule on the pancreas. Functional pancreatic NET may present at an early stage, and identification of the primary tumor may be challenging. B. Non-functioning pancreatic NET in the tail of the pancreas with associated hepatic metastases. Non-functioning pancreatic NET more typically present at a later stage, often as bulky lesions in the tail of the pancreas with associated liver metastases.

In general, in the absence of distant metastases or significant comorbidities, complete surgical resection of the primary tumor should be attempted [31,32]. The primary tumors in patients who are diagnosed due to symptoms of hormone hypersecretion may be occult (Figure 1A). In contrast, patients with non-functioning pancreatic NET are commonly diagnosed at a later stage (Figure 1B). The prognosis following surgical resection of localized NET is often excellent. Isolated insulinomas, for example, are generally treated with enucleation; long-term survival following surgery in this patient population exceeds 90% [33]. The role of surgical resection in patients with MEN1 syndrome remains more controversial because of the risk of additional tumors within the remaining pancreas and elsewhere [34,35].

In contrast to patients with pancreatic adenocarcinoma, hepatic resection may be beneficial in patients with metastatic pancreatic NET. Resection may be performed to render the patient free of macroscopic disease and to diminish systemic symptoms. Hepatic resection is generally favored in patients with limited hepatic disease. In a study of 170 patients, hepatic resection improved symptoms in over 90% of cases [36]. Debulking surgery in patients with more advanced disease may be recommended in selected patients if the majority (i.e., >90%) of the tumor burden can be resected [37]. The median survival for patients treated with this approach has been reported to be 7 years [38]. The reported survival rates for this surgical approach have been in excess of 60% at 5 years, which is twice that of patients with untreated liver metastases [38,39]. An important observation is that the survival of patients who have palliative hepatic debulking by 90% is indistinguishable from those who have complete resection (i.e., resection of all visible hepatic tumors) [39]. Also, the survival with this approach is the same among patients with functional and non-functional tumors [39]. Thus, surgical resection should be considered to potentially improve outcomes even though surgery may not be curative.

## Liver-directed Therapy

In patients with hepatic metastases who are not candidates for surgical resection, hepatic arterial embolization may be an appropriate palliative technique, provided that their disease is primarily confined to the liver, the portal vein is patent, they have not undergone a Whipple procedure (or pancreaticoduodenectomy), and that patients have an otherwise preserved performance status [40-42]. Response rates are generally >50%, whether measured by reduced hormone secretion or radiographic regression [40-43]. A variety of techniques have been employed, including bland embolization, chemo-embolization, embolization with chemotherapy eluting beads, or embolization using radioisotopes. There are currently no data confirming superiority of any one of these techniques over the others.

Hepatic metastases can also be treated with percutaneous or laparoscopic radiofrequency ablation (RFA) and cryoablation, either alone or in conjunction with surgical debulking [40,42]. While these approaches appear to cause less morbidity than either hepatic resection or hepatic artery embolization, the clinical benefit of this approach in patients with asymptomatic, small-volume disease has not been clearly established. Ablative techniques should be considered only in carefully selected patients [40,42,44,45].

## Cytotoxic Chemotherapy

Although carcinoid and pancreatic NET appear histologically similar, there is increasing evidence that pancreatic NET are more responsive to chemotherapy than are carcinoid tumors (Table 2). In an initial randomized trial, the combination of streptozocin and doxorubicin was associated with a combined biochemical and radiologic regression of 69%, as well as a survival benefit when compared to streptozocin and fluorouracil [46]. The median overall survival duration for patients treated with streptozocin and doxorubicin was 2.2 years. Streptozocin was subsequently approved by the FDA as a treatment for patients with pancreatic NET. The very high response rates reported in this study were derived in part from the historical use of non-standard response criteria. A large retrospective analysis of 84 patients with either locally advanced or metastatic pancreatic endocrine tumors showed that a three-drug regimen of streptozocin, 5-FU, and doxorubicin was associated with an overall response rate of 39% and a median survival duration of 37 months [47].

**Table 2:** Selected Trials of Cytotoxic Chemotherapy in Advanced Pancreatic NET

Regimen	Patients	Tumor Response Rate (%)	Median Progression- Free Survival	Median Overall Survival (Months)	Reference
**Prospective Studies**					

Chlorozotocin	33	30	17*	18.0	Moertel et al. 1992 [46]

Fluorouracil + Streptozocin	33	45	14*	16.8	Moertel et al. 1992

Doxorubicin + Streptozocin	36	69	18*	26.4	Moertel et al. 1992

DTIC	50	34	NR	19.3	Ramanathan et al. 2001 [67]

Temozolomide+ Thalidomide	11	45	NR	NR	Kulke et al. 2006 [52]

Temozolomide+ Bevacizumab	17	24	8.6	NR	Kulke et al. 2006 [51]

Temozolomide+ Everolimus	24	35	NR	NR	Kulke et al. 2010 [53]

**Retrospective Studies**					

Steptozocin+ Doxorubicin+ Fluorouracil	84	39	18	37	Kouvaraki et al. 2004 [47]

Temozolomide (diverse regimens)	53	34	13.6	35.3	Kulke et al. 2009 [49]

Temozolomide (single agent)	12	8	NR	NR	Ekeblad et al. 2007 [48]

Temozolomide+ Capecitabine	30	70	18	NR	Strosberg et al. 2010 [50]

NR: Not reported.

Despite the demonstrated efficacy of streptozocin-based regimens, their potential toxicity, together with a cumbersome 5 consecutive day infusion schedule, has precluded their more widespread use in patients with advanced pancreatic NET. Recent prospective and retrospective studies have suggested that oral temozolomide-based regimens may be at least comparable in efficacy to streptozocin-based regimens, and may also be more tolerable. In retrospective series, temozolomide-based therapy has been associated with overall response rates of 8-70% [48-50] (Table 2). Temozolomide has been evaluated prospectively in combination with thalidomide, bevacizumab, or everolimus, with overall response rates of 24-45% [51-53]. Neither the optimal dosing regimen for temozolomide, nor the relative activity of temozolomide as a single agent or in combination with other therapeutic agents has been clearly established.

## Somatostatin Analogs and Peptide Receptor Radiation Therapy (PRRT)

More than 90% of NET express somatostatin receptors, and somatostatin analogs (SSAs) have been shown to be effective in reducing symptoms of hormone hypersecretion associated with both carcinoid and pancreatic NET. In patients with midgut carcinoid tumors, treatment with the somatostatin analog octreotide has been shown to improve time to tumor progression over placebo. Ongoing studies are currently exploring whether somatostatin analogs have a similar antiproliferative effect in patients with pancreatic NET.

The high rate of somatostatin receptor expression in pancreatic NET also provides a rationale for peptide receptor radionuclide therapy in patients with inoperable or metastatic disease. The most frequently used radionucleotides for targeted radiotherapy in NET are yttrium (^90^Y), and lutetium (^177^Lu), which differ from one another in terms of emitted particles, particle energy, and tissue penetration[54,55]. Both the yttrium and the lutetium labeled compounds have demonstrated promising activity in NET patients. The radiolabeled somatostatin analog [^177^Lu-DOTA, Tyr^3^] octreotate, for example, has been utilized in the treatment of 504 patients with NET, and efficacy results, reported for 310 patients, suggest single agent activity [56]. Treatment with ^90^Y-DOTA tyr3-octreotide (^90^Y-edotreotide) was recently reported to be associated with high rates of symptom control, though only modest tumor response rates, in a prospective, phase II study [57]. Randomized studies comparing PRRT to treatment with "cold" octreotide are anticipated to better define the relative efficacy and toxicities associated with these regimens.

## Biologically Targeted Therapies for Pancreatic NET

Studies of biologically targeted therapies in pancreatic NET have, to date, focused primarily on inhibitors of the VEGF or mTOR signaling pathways. While objective RECIST-defined tumor response rates have been relatively low, recent studies have suggested that treatment with these agents is associated with improvements in progression-free survival.

### VEGF pathway inhibitors

Three tyrosine kinase inhibitors--pazopanib, sorafenib, and sunitinib--all with activity against VEGFR, have been evaluated in prospective trials of patients with advanced pancreatic NET. Pazopanib was evaluated in a prospective study enrolling 51 NET patients (29 with pancreatic NET and 22 with carcinoid) on stable doses of octreotide-LAR. Patients received pazopanib at a dose of 800 mg daily. The response rate among patients with pancreatic NET was 17%; no patients with carcinoid experienced a radiographic response (by RECIST) [58]. Sorafenib is another small molecule tyrosine kinase inhibitor with activity against VEGFR. In a study of 50 patients with carcinoid and 43 patients with pancreatic NET, preliminary analysis showed responses in 7% of the carcinoid patients and 11% of the pancreatic NET patients [59].

Sunitinib malate was evaluated in a multi-institutional phase II study enrolling 109 patients with advanced NET. Patients received repeated 6-week treatment cycles of sunitinib, administered orally at 50 mg once daily for 4 weeks, followed by 2 weeks off treatment [60]. Partial responses were observed in 2% of the carcinoid cohort and 16% of the pancreatic NET cohort. Based on evidence of activity in this study, an international randomized phase III study to confirm the activity of sunitinib in pancreatic NET was undertaken. The study was discontinued prior to a planned interim analysis after enrollment of 171 patients, 86 of whom received sunitinib and 85 of whom received placebo. The early discontinuation of the study precluded definitive hypothesis testing for differences in progression-free survival durations between the treatment and placebo groups. Nevertheless, analysis of the available data demonstrated that treatment with sunitinib was associated with a median progression-free survival (PFS) of 11.4 months, as compared with 5.5 months for placebo (*P*=.0001, Table 3) [61].

**Table 3:** Randomized Trials of Biologically Targeted Therapies in Pancreatic NET

Regimen	N (total)	Overall Response Rate	Median Progression-Free Survival/TTP	P value	Reference
Sunitinib (37.5 mg po qd) Placebo (+ best supportive care)	171	9% 0%	11.4 months 5.5 months	.0001	Raymond et al, 2011 [61]

Everolimus (10 mg po qd) Placebo (+ best supportive care)	410	5% 2%	11 months 4.6 months	<.0001	Yao et al, 2011 [65]

Everolimus (10 mg po qd) Everolimus (10 mg po qd) + Bevacizumab (10 mg/kg every other week)	CALGB 80701	(Ongoing)			

### mTOR Inhibitors

Tumor cell growth, proliferation, and apoptosis are regulated in part by a serine-threonine kinase called the mammalian target of rapamycin (mTOR). This enzyme also mediates downstream signaling from a number of pathways, including the VEGF and insulin-like growth factor (IGF) signaling implicated in NET growth. Temsirolimus and everolimus are rapamycin derivatives that have been evaluated recently in NET. Weekly intravenous administration of temsirolimus was associated with a response rate of 5.6% in one study of 37 patients with advanced progressive NET. Outcomes were similar between patients with carcinoid and pancreatic NET [62].

Everolimus was initially evaluated in a single-institution study, in which 30 patients with carcinoid tumors and 30 with pancreatic NET received doses of 5 or 10 mg daily plus depot octreotide (30 mg every 4 weeks). The overall tumor response rate in evaluable patients was 17% in carcinoid and 27% in pancreatic NET [63]. A follow-up multinational phase II study (RADIANT-1) enrolled 160 patients with advanced pancreatic NET and evidence of RECIST-defined progression following chemotherapy. In this non-randomized study, treatment with everolimus was associated with an overall response rate of 4.4% and progression-free survival duration of 16.7 months in those patients receiving octreotide. Among patients not receiving octreotide, the response rate was 9.6% and the progression-free survival duration was 9.7 months [64]. A subsequent phase III study randomized 410 patients with progressive advanced pancreatic NET (RADIANT-3) to receive treatment with everolimus or placebo; octreotide was given at the discretion of the investigator. This study demonstrated significant improvements in the primary endpoint of investigator-assess PFS associated with everolimus as compared to placebo (11 months versus 4.6 months, [*P *<.0001, Table 3]) [65]. The overall tumor response rate associated with everolimus in this study was 5%.

Ongoing studies are currently evaluating combinations of targeted agents in patients with advanced pancreatic NET. A combination of the mTOR inhibitor everolimus and the VEGF inhibitor bevacizumab was recently shown to be well tolerated and associated with antitumor activity (overall response rate 26%) in an initial phase II study enrolling patients with low or intermediate grade NET [66]. CALGB 80701 is currently randomizing patients with advanced pancreatic NET to receive either treatment with everolimus or everolimus + bevacizumab to assess the relative efficacy and toxicity of these regimens (Table 3).

## Conclusions

Patients with pancreatic NET present with diverse symptoms related to hormonal hypersecretion, tumor bulk, or both. Accurate diagnosis of this condition and differentiation of pancreatic NET from the more common pancreatic adenocarcinomas is a critical first step in developing an appropriate treatment plan. Similarly, pancreatic NET should be considered separately from carcinoid tumors, which arise in other sites. Surgical resection remains the mainstay of treatment for patients with localized disease. A number of treatment options are available for patients with advanced pancreatic NET. These include hepatic-directed therapies, including surgical resection and hepatic artery embolization. Systemic treatment options include the use of SSAs for control of hormonal hypersecretion, as well as alkylating chemotherapy. Recent studies have also reported that the tyrosine kinase inhibitor sunitinib and the mTOR inhibitor everolimus improved progression-free survival in patients with pancreatic NET, further expanding the therapeutic arsenal available to patients with this disease. Future studies will likely build on these results, further improving therapeutic options for patients with this disease.

## Competing interests

MK has served as a consultant for Pfizer, Novartis, Ipsen, Lexicon Pharmaceuticals, and Molecular Insight Pharmaceuticals. JB has no competing interests. LK has served as a consultant and/or received honorarium from Novartis, Pfizer, and Delcath. JP has received research funding and honorarium, and served as a consultant and as a speaker for Novartis and Pfizer. JY has served as a consultant for Ipsen, Novartis, and Pfizer.

## Authors' contributions

All authors were involved in drafting the manuscript and revising it critically for important intellectual content. All authors have also read and approved the final version of the manuscript to be published.
